# Dementia-related primary care training needs: a qualitative study

**DOI:** 10.1055/s-0045-1809544

**Published:** 2025-07-02

**Authors:** Tomas Leon, Deiza Troncoso, Soledad Barria, Magda Kaczmarska, Brian Lawlor, Andrea Slachevsky

**Affiliations:** 1 University of Chile, Hospital del Salvador, Faculty of Medicine, Memory and Neuropsychiatric Centre, Neurology Department, Santiago, Chile.; 2 Trinity College, Dublin, Ireland.; 3 Ministry of Health, Santiago, Chile.; 4 Universidad de los Lagos, Department of Family Medicine, Osorno, Chile.; 5 Foundation Dementia Action Alliance, Krakow, Poland.; 6 Geroscience Centre for Brain Health and Metabollism, Santiago, Chile.; 7 University of Chile, Faculty of Medicine, Neuropsychology and Clinical Neuroscience Laboratory, Department of Physiopathology, Biomedical Science Institute, Neuroscience and East Neuroscience Departments, Santiago, Chile.; 8 Universidad del Desarrollo, Clínica Alemana, Department of Medicine, Neurology Unit, Santiago, Chile.

**Keywords:** Dementia, Primary Health Care, Professional Training

## Abstract

**Background:**

Most guidelines recommend that people living with dementia and their care partners should be managed in primary care. However, the knowledge and confidence of these teams in managing dementia is low, and training programs are lacking.

**Objective:**

To identify the training needs of primary care teams by integrating insights from these professionals, as well as dementia patients and their care partners.

**Methods:**

Qualitative research methods were applied, using focus group interviews with health professionals and individual interviews with people living with dementia and their care partners. A direct qualitative analysis of 15 recorded interviews (3 focus groups and 12 individuals) was performed using the transcribed data.

**Results:**

Primary care professionals recognize the importance of continuous education on dementia and expressed the need for more knowledge about diagnosis, symptom management, and interpersonal and communication skills. Care partners and dementia patients highlighted the need for a better diagnostic disclosure process, improved continuity of care, and availability of greater postdiagnosis support.

**Conclusion:**

Our study, novel in Latin America, strongly supported the need for more training in dementia for primary care professionals, as well as for additional content and information not usually included in standard dementia education.

## INTRODUCTION

The prevalence of dementia in Chile is 1.06% of the population, which means that, currently, over 200,000 individuals are living with dementia in the country. 1 It is estimated that over half a million people will have dementia in Chile by 2050. 2 In 2017, the Chilean Ministry of Health launched the National Plan of Dementia (NPD) in 3 specific areas, including the capital and other regions, through the public health system. The NPD was complemented by the Explicit Guarantee System (Garantias Explicitas en Salud, GES), in 2019, across the whole country. The GES provided a list of legally guaranteed interventions and pharmaceutical and nonpharmaceutical treatment options based on a diagnosis in the public health system

In line with international guidelines, 3 4 5 NPD and GES require that both specialists and primary care providers develop the capacity to diagnose and treat people living with dementia (PD), with the latter having a critical role in dementia diagnosis and care both in the NPD and the GES. This is in line with evidence that a collaborative model focusing on primary care improves outcomes such as quality of life, behavioral symptoms, care partner burden, medical services, and health costs. 6 7

Despite having a strong and well-developed system, 8 the level of knowledge and confidence among primary care teams (PCTs) in Chile on dementia diagnosis and care remains low. 9 Although the NPD included a plan to introduce dementia training programs and to develop continuous support from secondary care, these were created without considering the PCT's voice, as well as PD and their care partners' (CP) voices.

Amplifying PCT's agency and knowledge during and after diagnosis could improve the continuity of care for PD and CP, resulting in improvement in quality of life and possibly a reduction in hospitalization. 10 Training for PCT should always consider patients' context, time constraints in assessment, lack of resources, and a person-centered model of primary care. 11

In recent years, efforts have been made to include the voices of PD and their CPs in professional education, clinical implementation, and research. 12 Previous research from Ireland suggest that this improves the care process, by tailoring the interventions and research according to the patients' needs. Furthermore, this improves adherence, efficacy, and relevance of treatment and research. 13 14 Nevertheless, to the best of our knowledge, this methodology has not been applied in LA despite the relevance of primary care in the health system. 15

The overarching goal of this study was to assess the theoretical, practical, attitudinal, and resource needs of PCT in diagnosing, treating, and supporting PD and their CPs, as well as to explore the expectations of those individuals regarding care provision. These insights were used to design an optimal training course for PCT.

## METHODS

We gathered information on the needs of PCT, complementing it with the needs and priorities of people with dementia and their care partners using qualitative methods, following protocols used internationally. We selected this methodology to tailor the primary care training course program to the specific needs of the care providers and the care recipients.

In this study, the type of sampling was nonprobabilistic and intentional. The areas to investigate were selected to gather different opinions and experiences from participants who have received the training from the NPS and others who have not. Among areas not included in the NPD pilot, we recruited participants from the country's capital, representing areas with different socioeconomic realities and others from areas outside the capital.

The distribution of the sample and the applied techniques are found in Table 1 .

**Table 1:** Distribution of participants and technique description

Units of analysis/Technique	Areas w/o NPD training from the capital: *Pedro Aguirre Cerda (PAC) and Vitacura*	Areas with NPD training outside the capital: *Copiapó, San Felipe, Los Andes and Putaendo*	Areas with NPD training: *Peñalolén, Punta Arenas y Osorno*
PCT / Focus group	CESFAM: Amador Neghme (GP, Ocupational therapist, Clinical Psychologist) y Vitacura (GP, Ocupational therapist and social worker). Date: January 25, 2022	CESFAM: Candelaria del Rosario (GP, 2 Social workers, Clinical Psychologist), Segismudo Iturra (Speech therapist), Centenario (Clinical Psychologist), Valle los Libertadores (GP). Date: January 20, 2022	CESFAM: La Faena (GP, Ocupational therapist and social worker), 18 de Septiembre (Médico, physical therapist), Mateo Bencur (Nurse), Marcelo Lopetegui (2 family doctors, Ocupational therapist, physicial therapist, 2 nurses). Date: January 18, 2022
PD / Individual interview	1 patient from PAC and 1 from Vitacura. Date: March 1–4, 2022	1 patient from Los Andes and 1 patient from Copiapó. Date: January 28 ^th^ and February 2 ^nd^ , 2022	1 patient from Peñalolén and 1 patient from Punta Arenas. Date: February 2 ^nd^ and March 2 ^nd^ , 2022
CP / Individual interview	2 care partners from PAC and Vitacura. Date: March 1–4, 2022	2 care partners from Copiapó and Los Andes. Date: January 28–31, 2022	2 cuidadoras Punta Arenas y Peñalolén. Date: January 31 ^st^ and February 2 ^nd^ , 2022

Abbreviations: CESFAM, primary care center; CP, care partners; GP, general practitioner; NPD, National Plan of Dementia; PCT, primary care teams; PD, people diagnosed with mild dementia.

### Data collection

Information was collected according to the following criteria: First, PCT members who had received the online and in-person training from the NPD and had more experience caring for PD, called “expert” teams. The APS-GES 85 teams correspond to PCT from 2019 who take care of PD but have not received training. Second, PD, defined as people diagnosed with mild dementia. Finally, CPs who were self-classified as informal care partners. They are also called family caregivers or care partners, meaning those who provide care to family or friends, usually without payment.

All participants signed an informed consent form. The study was conducted in accordance with the Declaration of Helsinki and approved by the Committee of the Facultad de Medicina, Universidad de Chile, protocol code 292–2020, on 8 June 2021.

Data collection happened between the months of January and March 2022, through 2 approaches: focus groups and individual semi-structured interviews.

### Data collection approaches

The questions included in the focus groups and interviews were translated and adapted from the Prepared project performed in Ireland. 16 The digital records of both approaches were retained in a file to which only the researchers of the study had access.

Focus groups were carried by Zoom Workplace (Zoom Communications Inc.) between January and March 2022.

The questions used in the study are available in **Supplementary Material Table S1** (available at https://www.arquivosdeneuropsiquiatria.org/wp-content/uploads/2025/04/ANP-2025.0008-Supplementary-Material.docx ). Adaptations from the Prepared project 16 were supplemented with questions proposed by the research team to assess the practical resource needs, interpersonal training requirements of PCTs, and the expectations of people living with dementia and care partners.

### Data analysis

The transcribed data were analyzed using direct content analysis. 17 This approach takes the literal rather than latent meaning of what is studied.

The procedure consists, first, of classifying the information according to the units of analysis, that is, each of the speakers or cases. Next was the reflective reading by the research team of the transcripts. Then, we established categories inductively, following the study's objectives, which were guided by the questions formulated in the interviews and focus groups.

The type of coding used was open, considering the total number of paragraphs, sometimes more than one because the expressed idea was completed, assigning the codes and rewriting them as the material was reviewed.

After that, we grouped the coded texts (“cutting and pasting”) to carry out a thematic structuring of the analysis.

This classification allowed us to propose specific topics and content to be considered in the training.

## RESULTS

The results are categorized under the following headings: PCT, CPs, and PD. The main themes emerging from the focus group and individual Zoom interviews are presented below:

### Primary care teams

The main themes from the content analysis for the PCTs were:

#### Training

The level of preparation of the teams to carry out a comprehensive approach to dementia is insufficient to meet the demands placed on their practice, especially among teams that were not trained in the NPD pilot.

I would also like to have the security, and for the peace of mind of the families, to be able to diagnose, finally, with total certainty, that I know that in dementia it is not so easy, but I do not know, more empowered in the medical part. So, on super simple issues concerning dementia, the subtypes, everything that has to do with the cognitive-behavioral symptoms of dementia, everything that has to do with non-pharmacological interventions for these patients. It is essential that we continue to receive or participate in these trainings.

Additionally, PCTs feel unprepared to provide psychoeducation to care partners.

I believe that one of the main problems has to do with the education of care partners because, in general, this generates a lot of stress, a lot of anxiety, and we are not prepared to do that. Not only because of what care implies, as would happen with a bedridden patient, but also because of the cognitive deterioration that is seen in the person and that the family member perceives it and often does not understand it.

#### Behavioral symptoms

Alongside difficulties and inconsistencies in the diagnosis of dementia, PCTs face challenges in postdiagnostic support and treatment of dementia. The teams from the NPD pilot study mention they feel incapable of suggesting effective approaches to address behavioral changes experienced by people with dementia.

For me, as a doctor, the biggest challenge is managing behavioral maladjustment because that totally destabilizes the person, the team, the family, the neighbors, everybody. There are also problems generated by patients who start their treatment in private care with a specialist and then, due to financial issues, they must continue their treatment in primary care with limited access to specialists and medications.

Additionally, PCTs recognize that they currently lack sufficient information and access to additional resources to support care partners, particularly access to non-pharmacological interventions for people with dementia. As stated by one of them: “non-pharmacological interventions, we don't have any to offer. The gender component in the care issue would also be good”.

#### Non-clinical communication and interpersonal skills regarding the dementia diagnosis

This refers to the interpersonal and ethical aspects involved in the progression of the disease in the person, their care and management of autonomy. Some participants admitted they struggle to find the best way to share the dementia diagnosis with patients.

Furthermore, others believe medical teams are still lacking in how they deal with the issues of rights, discrimination, and how to properly address patients. This also includes the need for person-centered education among all PCTs that reflect a human rights and disability-centered ontology for PD. 18

### People with dementia

The main themes from the content analysis for the PD were:

#### Diagnosis disclosure

Among the PD that were interviewed, when asked about the circumstances of their diagnosis, most did not recall the specifics.

As the interview progressed, one interviewee who did not initially indicate knowing her diagnosis related her reflections on the delivery of the diagnosis, indicating that to her, it is necessary to have the diagnosis explained simply.

#### Postdiagnosis support

People with dementia indicated that the information about their diagnosis and the treatment is not addressed to them but rather to the CP, which makes them feel excluded from the process. One of the participants stated: “No, not that, because I go with my daughter, and she is the one who always talks to the doctor.”

### Care partners

The main themes from the content analysis of the care partners responses were:

#### Continuity of care

One aspect mentioned by all the CPs interviewed was the continuity of care of the professional team that attends to their care recipient, specifically the primary physician.

An additional complexity is faced by families who begin the diagnostic process in the private health care system. Within that system, families move from one specialist to another without any clinical cross-communication or coordination, despite pharmacological treatment being recommended. As a result, the family CPs are left on their own to coordinate and advocate for the PD, while simultaneously lacking the training or understanding of potential contra-indications or serious medical issues that may arise because of this lack of care coordination.

It would be ideal for a single doctor to see it. (...) Because he did the test, all those things, but ideally, a single doctor would see it because one goes to a doctor, and another attends him, and you must start from the beginning. Furthermore, they are supposed to have their medical record. They know everything.

#### Carer burden

Finally, CPs mentioned the emotional burden of caring for a PD and the lack of support they receive from the existing system in managing their own mental health.

So, these are things that you have to try to understand, to address, to internalize. That the changes are like that. Moreover, well, work on that anger issue, of my sorrow, not to turn it into anger, because that is what I realize. Moreover, suddenly I am screaming or angry because I tell him the same thing three times, and he does not understand the instruction...

## DISCUSSION

In the present study, we sought the views of PCTs, PD, and CPs to identify what should be included in an online dementia training program. To our knowledge, our study is the first Latin American research that identifies the specific training needs in dementia care and includes the voices of PD and their CPs.

The main finding from this qualitative study is that participants identify technical and interpersonal topics that should be included in training schemes. While the PCT needs to be focused on diagnosis, management of behavioral and psychological symptoms of dementia (BPSD), and interpersonal and communication skills around the condition, PD and their CPs highlighted the importance of diagnosis disclosure, continuity of care, and addressing CP burden. Individuals with dementia also noted that they are overlooked in the diagnostic and diagnostic disclosure process, usually not being considered in the interview.

The study also showed a lack of coordination between the different parts of the health care systems in Chile, including between public and private care, as well as between primary and secondary care. This was mentioned by both PCT and CPs. In some cases, private or secondary care perform interventions, such as diagnosis or pharmacological treatments that are not continued due to either monetary issues or lack of coordination with the rest of the health care network.

Dementia care in Chile has experienced changes, starting with the pilot of the NPD, followed by the inclusion of dementia in the GES. This has generated inequities so that some areas have a complete care network, including daycare centers, memory units, and a trained PCT, while others lack one or more aspects of these care networks. Those inequities have also been described across other Latin American countries. 19

Interestingly, PD note that they are often overlooked in the care process. This is in line with previous reports in the literature from individuals with brain or mental disorders, and it is referred to as epistemic injustice. 20 This could arise from a lack of person-centered training and approaches among PCTs. 21

Although our sample was small, we included people from different location of Chile, including different economic, cultural, and social realities. It is very interesting that the opinions of CPs and PD did not vary much despite their socioeconomical status. One possible explanation is that, despite their context, they are all from the public health system. However, our clinical experience indicates that those needs are not fulfilled regardless of the socioeconomical context.

Our results are in line with those reported by Foley et al., 22 who used qualitative interview methods with primary care doctors, PD, and care partners in Ireland, identifying five distinct educational needs, both clinical and nonclinical, emerging from the interviews, namely diagnosis, disclosure, signposting of local services, counselling, and management of BPSD. The primary care doctors highlighted educational shortcomings in diagnosis, disclosure, and BPSD, whereas PD and their CPs reflected educational needs in counselling. 22

It has been reported that training programs have not had a real impact on practitioners and the quality of care provided. 23 One possible reason for the small impact of training programs is that most of the courses have been conducted by specialists (e.g., STAR-E 24 ) who, despite their expertise, are removed from the complexities and requirements of dementia training in PC and, therefore, do not fulfil the PCT training requirements. 16

As a result of the information in this study, we have created a new curriculum for an online training course on dementia for PCTs, which is currently in development and implementation. As recommended by our participants, in this course we have included not only “classical” clinical training but also information focused on ethical knowledge and interpersonal skills. To improve care continuity, we have included information on healthcare system dynamics and referral protocols to ensure better communication between caregivers. We have also added a module on interpersonal skills, team self-care and how to interact with and relate to people with cognitive impairment. The posttraining evaluation of the course could bring important insights into the impact of the proposed strategies.

The main strength of our study is the inclusion of PD and their CPs. Interviewing the main beneficiaries of PCT provides local lived experience information and contributes to knowledge on aspects that are not typically considered by the health team. It is also a major shift in the way research about dementia is performed in Chile and, more broadly, Latin America, placing patients at the center and considering them as active participants in generating new knowledge and information.

The main weakness of the study is the relatively small number of interviews, which may have resulted in an incomplete view of the training needs of PCTs. It is possible that specific groups, such as the indigenous population, are not well represented in our sample. Those populations might have special characteristics and needs, as has been described by Furlano et al. 25 However, we attempted to mitigate this weakness by including participants from different contexts across the country, so the study's design and numbers are similar to those used by Foley et al. 22

In conclusion, consistent with what is seen worldwide, 3 5 there is a major need for dementia training in primary care in Chile. The integrated and comprehensive model that can be delivered in primary care is ideal to manage and support chronic and progressive diseases like dementia. However, PCTs need training and support to do it properly. 5

The present study has identified the training needs and components that should be included in developing a dementia training program. It is worth noting that these are not generally considered in training designs by experts. The next steps are the implementation and evaluation of this online training program across Chile.
